# The Use of Artificial Intelligence to Predict the Prognosis of Patients Undergoing Central Nervous System Rehabilitation: A Narrative Review

**DOI:** 10.3390/healthcare11192687

**Published:** 2023-10-06

**Authors:** Min Cheol Chang, Jeoung Kun Kim, Donghwi Park, Jang Hwan Kim, Chung Reen Kim, Yoo Jin Choo

**Affiliations:** 1 Department of Rehabilitation Medicine, College of Medicine, Yeungnam University, Daegu 42415, Republic of Korea; wheel633@ynu.ac.kr; 2 Department of Business Administration, School of Business, Yeungnam University, Gyeongsan-si 38541, Republic of Korea; kimjk70@yu.ac.kr; 3 Department of Rehabilitation Medicine, Daegu Fatima Hospital, Daegu 41199, Republic of Korea; bdome@hanmail.net; 4 Department of Rehabilitation Technology, Graduate School of Hanseo University, Seosan, Chungcheongnam-do 31962, Republic of Korea; profpo@naver.com; 5 Department of Physical Medicine and Rehabilitation, Ulsan University Hospital, University of Ulsan College of Medicine, Ulsan 44033, Republic of Korea; crkim@uuh.ulsan.kr

**Keywords:** artificial intelligence, central nervous system, rehabilitation, prediction, prognosis

## Abstract

Applications of machine learning in the healthcare field have become increasingly diverse. In this review, we investigated the integration of artificial intelligence (AI) in predicting the prognosis of patients with central nervous system disorders such as stroke, traumatic brain injury, and spinal cord injury. AI algorithms have shown promise in prognostic assessment, but challenges remain in achieving a higher prediction accuracy for practical clinical use. We suggest that accumulating more diverse data, including medical imaging and collaborative efforts among hospitals, can enhance the predictive capabilities of AI. As healthcare professionals become more familiar with AI, its role in central nervous system rehabilitation is expected to advance significantly, revolutionizing patient care.

## 1. Introduction

Recently, artificial intelligence (AI) has been applied in various industries such as healthcare, finance, and manufacturing, and many studies have been conducted to implement AI to overcome the limitations of existing traditional methods for analyzing data and obtaining meaningful results [1,2]. Machine learning is eliciting growing interest and is increasingly applied extensively in healthcare. Deep learning techniques, particularly convolutional neural networks (CNNs), are making significant strides in medical image analysis, having been effectively deployed for image segmentation, object detection, feature extraction, among other tasks [3]. The technology has proven to be highly valuable for early disease detection and the assessment of the size and rates of complications through the analysis of images, including MRI, computed tomography (CT), and ultrasound images [4,5,6]. Furthermore, advancements in real-time image analysis can aid surgical procedures, especially by enhancing surgeons’ precision and decision making [7]. AI can also be applied to follow-up measures and monitoring, thus enabling clinicians to track changes more easily and adjust treatment plans as needed [3]. Moreover, it has enabled remote healthcare services, which benefit underserved areas and mobility-challenged patients [8]. Similarly, several studies in the field of rehabilitation medicine have integrated it into clinical practice [9].

Rehabilitation medicine for patients with central nervous system (CNS) disorders aims to improve the function and quality of life of individuals with physical disabilities [10]. In rehabilitation medicine, physicians must accurately assess the prognosis of each patient and establish treatment goals. Based on these goals, appropriate rehabilitation strategies could be appropriately elucidated [11]. For patients with CNS disorders, factors such as lesion size, lesion location, and demographic data are traditionally used to determine the prognosis [12]. In addition, special tools such as diffusion tensor tractography and transcranial magnetic stimulation can be used to evaluate therapeutic prognosis [13]. However, these methods often do not consider multiple variables and tend to rely only on limited factors. Diffusion tensor tractography and transcranial magnetic stimulation have high false-positive and false-negative rates [14]. Moreover, the prognosis obtained through these conventional methods only shows general trends and does not predict personalized treatment outcomes [15].

Currently, research is being conducted to explore whether AI can help predict the prognosis of patients receiving rehabilitative treatment. Here, we briefly summarize the history of advancements in AI and review its utilization in predicting the prognosis of patients receiving CNS rehabilitation. 

## 2. History of Artificial Intelligence

The concept of AI was established in 1943, when neurosurgeon Warren McCulloch and logician Walter Pitts published a paper titled “A Logical Calculus of Ideas Immanent in Nervous Activity” proposing the creation of artificial neurons based on the fundamental principles of human neurons, which operate like on–off switches and connect them in a network-like structure to mimic simple human functions [16]. Because of the “all-or-none” character of nervous activity, neural events and their relations can be addressed using propositional logic [16].

In 1950, Alan Turing published a paper titled “Computing Machinery and Intelligence” [17]. The importance of the paper is reflected in analyzing the feasibility of creating thinking machines, posing the question “Can machines think?”. Additionally, he introduced the “Turing test,” an experiment aimed at determining whether a machine possessed AI. The Turing test was based on the idea of preparing a questioner and two respondents, where one respondent was a computer and the other was a human. The questioner is a human who was unaware of which respondent was the computer. Responses were conveyed solely through a keyboard, and if the questioner could not consistently distinguish which respondent was the computer, the computer was considered to have AI [17].

The term “AI” was first coined in 1956 by John McCarthy, who organized the Dartmouth conference and invited ten scientists [18]. During this conference, the term AI was first used, and McCarthy defined the concept of AI as “the science and engineering of making intelligent machines” [18]. 

In 1958, the neurobiologist Frank Rosenblatt devised an artificial neural neuron “perceptron”, inspired by interconnected neurons in the human brain, aiming to train computers using multiple neural networks, such as the human brain [19]. The perceptron algorithm, which aggregates multiple inputs into a single output, is an early form of artificial neural networks and remains one of the most commonly used models. However, in 1969, Marvin Minsky and Seymour Papert demonstrated the limitations of the perceptron [20]. Thereafter, skepticism about the progress of AI technology spread, and research on AI entered a period of stagnation. In 1986, Geoffrey Hinton developed the “multilayer perceptron” model, which overcame the limitations of the original perceptron [21]. In 2006, Hinton developed a deep neural network (DNN) algorithm [22]. Using a new function called a rectified linear unit (ReLU) as an alternative to the previously used sigmoid function, the DNN solved the vanishing gradient problem. In addition, using the dropout function, which deactivates neurons randomly during training, helps mitigate the issue of overfitting by preventing the learning process from becoming overly biased towards the training data. Subsequently, with the advancement of computer technology and the explosive growth of information, AI technology has rapidly developed and is currently applied to almost every social and scientific domain, reshaping human life.

## 3. Use of AI to Predict the Prognosis of Patients Undergoing CNS Rehabilitation

We explored instances of AI applications for predicting the prognosis of patients who have undergone CNS rehabilitation. Twelve articles [23,24,25,26,27,28,29,30,31,32,33,34], categorized as stroke, traumatic brain injury (TBI), and spinal cord injury (SCI), were included in this review. Details of the included studies are presented in Table 1.

### 3.1. Use of AI to Predict the Prognosis of Patients with Stroke

Gupta et al.’s study published in 2017 [23] used AI to predict motor outcome in patients with stroke. They recruited 575 patients with intracerebral hemorrhage and collected more than 200 data points, including demographic data, laboratory results, state at admission, treatment, neurological defects, hospital complications, medical history, and discharge data. Random forest and linear regression models were used to develop an AI algorithm. Additionally, they employed a backward elimination approach to eliminate unnecessary variables, resulting in predictive models that utilized six and four variables for motor function prediction. They categorized 3- and 12-month outcomes as either ‘good’ (Modified Rankin Scale: 0–3) or ‘poor’ (Modified Rankin Scale: 4–6) based on functional status. The areas under the curve (AUCs) were 0.89 and 0.87 for predicting 3-month and 12-month motor outcomes, respectively. The high accuracy of the AI models can be attributed to their utilization of a more precise scoring system in algorithm development by integrating existing cognitive and physical function assessment tools.

Subsequently, many studies have been conducted to develop AI algorithms for predicting prognosis after stroke [24,25]. Various data collected in the early stages after stroke onset, such as age, sex, smoking, laboratory findings, comorbidities, modified Rankin Scale score, Barthel index, oral intake, nutritional state, activities of daily living, Berg balance test score, gait speed, 6-min walk test score, Fugl–Meyer assessment score, Mini-Mental State Examination score, and language function, were used as input data. To develop the AI algorithms, various models, including logistic regression, decision tree, random forest, support vector machine, extreme gradient boosting, deep neural network (DNN), adaptive boosting, and K-nearest neighbors, were used. The output of the developed AI algorithm was the motor outcome, which was categorized according to Barthel index scores or the modified Rankin Scale at discharge. The developed AI algorithms achieved AUCs predominantly ranging from the late 0.7s to the late 0.8s [24,25]. 

In addition, in 2022, Kim et al. [26] attempted to develop a practical AI prediction model using a small amount of input data, which are commonly checked in almost all stroke hospitals. The following demographic and clinical data were collected during the early stages of stroke: age; sex; type of stroke (ischemic/hemorrhagic); modified Brunnstrom classification (MBC); functional ambulation score (FAC); and Medical Research Council (MRC) score for muscle strength of the shoulder abductor, elbow flexor, finger flexor, finger extensor, hip flexor, knee extensor, and ankle dorsiflexor of the affected side. They used data from 833 consecutive patients with stroke. Patients with an MBC of <5 and FAC of <4 at 6 months after stroke onset were considered to have a “poor” outcome, whereas those with an MBC ≥ 5 and FAC ≥ 4 were considered to have a “good” outcome. In the model developed using the DNN, the AUC was 0.836 for upper and lower limb motor functions. The input variables used by Kim et al. (2022) [26] are data commonly collected to assess the functional status of patients with stroke across various institutions. Compared with prognostic prediction models developed using a more extensive clinical dataset, it demonstrated outstanding performance in terms of accuracy. These results indicate the potential generalizability of predictive models developed using common variables across many institutions.

Although most previous studies used demographic or clinical data as input variables for developing AI algorithms to predict prognosis after stroke, some recently used imaging data as input variables for the development of AI algorithms. In 2021, Kim et al. [27] used three consecutive T2-weighted axial brain magnetic resonance (MR) images at the level of the corona radiata per patient from 221 patients with a corona radiata infarct and created an AI model to predict ambulatory outcomes at 6 months after the infarct. They used a CNN, and the AUC of the developed algorithm was 0.751. To increase the prediction accuracy, Kim et al. combined clinical data. The AUC was significantly improved to 0.919. In 2022, Shin et al. [28] developed an AI algorithm using brain MR image data, not only from the corona radiata infarct but also from all patients with stroke. They obtained whole T2-weighted axial brain MR images of each patient taken at an early stage of stroke from 1233 patients with stroke. Favorable outcomes in the upper and lower limbs were categorized as having MBC scores of ≥ 5 and FAC scores of ≥ 4, respectively, at 6 months after stroke, and poor outcomes were defined by MBC scores of < 5 and FAC scores of < 4. The CNN architecture was employed to train the image dataset. For the prediction of upper and lower limb motor functions using the validation dataset, the AUC were determined to be 0.768 and 0.828, respectively. Furthermore, the sensitivities were 71.36% and 78.95%, respectively, and the specificities were 71.14% and 79.61%, respectively.

While previous studies have demonstrated the potential of AI in predicting functional outcomes in stroke patients, its accuracy is not yet sufficiently high for practical use in real-world settings. AI algorithms utilize limited or structured data, which may impede their performance. The accuracy of machine learning networks, especially DNNs, and data quantity are positively correlated [35]. However, there are constraints associated with obtaining structured data that are commonly collected in clinical settings, such as demographic and clinical data. Stroke, which is prevalent among the elderly population, is often accompanied by comorbidities [36]. This can introduce confusion when developing predictive algorithms that solely target stroke because the presence of comorbidities must be considered. Consequently, collecting abundant data exclusively from patients with stroke can be challenging. Image data can be leveraged to enhance the algorithm performance. Most patients with stroke undergo brain MRI or CT scans for diagnostic purposes [37]. Combining stroke lesion images with clinical data can facilitate the development of robust AI algorithms.

Previous studies have employed various AI algorithms to develop predictive models for stroke outcomes, including random forests, decision trees, logistic regression, support vector machines, DNNs, CNNs, extreme gradient boosting, adaptive boosting, and k-nearest neighbors. Random forest and decision trees enhance the accuracy and address diverse datasets through ensemble learning [38]. Logistic regression, a simple linear classification algorithm used for binary and multiclass classification tasks, provides interpretable results [39]. Support vector machines are effective at classifying data with a maximum margin [39]. DNNs and CNNs are well-suited for learning complex data patterns and performing tasks such as image and speech recognition [35,38]. Extreme gradient boosting and adaptive boosting facilitate model performance optimization and error minimization [39]. K-nearest neighbors are useful for classification and regression tasks based on data point proximity in the feature space [39].

### 3.2. Use of AI to Predict the Prognosis of Patients with Traumatic Brain Injury

In 2016, Rizoli et al. [29] recruited 1089 patients with TBI. They used several clinical data, including age, sex, systemic blood pressure, Glasgow Coma Scale, Head Abbreviated Injury Scale, Marshall score on the first head computed tomography (CT), and pupil reactivity at emergency department admission as input data. Outcome was categorized into acceptable outcome (Glasgow outcome scale > 4 at 6 months after onset) and poor outcome (Glasgow outcome scale ≤ 4 at 6 months after onset). They used a decision tree to create an algorithm for predicting the prognosis. The decision tree had a sensitivity of 72.3%, a specificity of 62.5%, and an AUC of 0.67. The poor performance of the prediction model can be attributed to various reasons, including TBI’s characteristic wide-ranging outcomes and the nature of decision trees. Decision trees can exhibit instability when predicting new data, and the selection of criteria values at each step of forming a tree structure plays a crucial role [40]. Such low-performance models are difficult to apply in actual clinical settings. Utilizing more patient data or applying various recently developed deep learning algorithms may enhance the prediction accuracy.

In 2020, Gravesteijn et al. [30] used clinical data from 11,022 patients with TBI and compared the capacities of machine learning and traditional regression to predict patient prognosis. They used age; Glasgow Coma Scale score; initial CT findings; presence of subarachnoid hemorrhage and hypoxia; and blood levels of glucose, sodium, and hemoglobin. They divided the patients’ outcomes at 6 months after onset into favorable outcome (Glasgow outcome scale ≥ 4) and unfavorable outcome (Glasgow outcome scale <4). The ML algorithms were developed using support vector machines, random forests, gradient boosting machines, and a DNN. The average AUC was 0.82, which is not significantly different from that of the traditional regression test. Ultimately, this comparison proved that the key to improving prediction accuracy is to incorporate predictive variables with a substantive incremental prognostic value because using new ML algorithms did not improve outcome predictions. In addition, ongoing refinement is necessary to ensure that the developed algorithms can be applied to emerging populations.

In the same year, Matsuo et al. [31] studied the feasibility of machine learning for predicting poor in-hospital outcomes (Glasgow Outcome Scale < 4) after TBI. They included 232 patients and the following clinical data were used as inputs: age, Glasgow Coma Scale score, abnormal pupillary response, systemic blood pressure, major extracranial injury, CT findings, and laboratory findings (glucose, C-reactive protein, and fibrin/fibrinogen degradation products). They used ridge regression, least absolute shrinkage and selection operator (LASSO) regression, random forest, gradient boosting, extra trees, decision trees, Gaussian naïve Bayes, multinomial naïve Bayes, and support vector machines to create the AI algorithm. Random forest showed the best performance for poor-outcome prediction, with 100% sensitivity, 72.3% specificity, 91.7% accuracy, and an AUC of 0.895. The developed random forest model may be useful for predicting adverse outcomes in patients with TBI. However, it is important to note that a relatively small sample size was used, and important parameters, such as hypoxia or anemia, were not considered. Additionally, approximately half of the subjects had severe TBI; therefore, caution should be exercised when applying the model to patients with mild TBI. In the future, the development of prediction models categorized according to injury severity will be necessary.

Previous AI studies predicting the prognosis of patients with TBI divided the functional outcomes using the Glasgow Outcome Scale. However, the Glasgow Outcome Scale classifies patients’ functional state into death, neurovegetative stage, severe and moderate disability, and good recovery [41]. Thus, it is necessary to utilize tools that allow for a finer measurement of the function of patients with TBI to create more precise AI models. Consideration could be given to the use of the Glasgow Outcome Scale-Extended, which provides a more comprehensive and detailed assessment of the functioning of patients with TBI, or the functional independence measure, which evaluates cognitive and physical independence. Another approach is the use of cutting-edge technologies to gather precise data. This may entail considering the use of AI-based wearable devices, such as wearable accelerometers, that are capable of capturing motion data and providing insights into a patient’s level of physical activity, gait, and balance [42]. Alternatively, an electroencephalogram can be used to monitor brain activity and detect anomalies, thus serving as a means of collecting detailed and accurate data [43].

### 3.3. Use of AI to Predict the Prognosis of Patients with Spinal Cord Injury

In 2016, Zariffa et al. [32] defined predictive values for the impairment assessment of simultaneous functional tasks in traumatic cervical SCIs as measured by the Graded Redefined Assessment of Strength, Sensibility, and Prehension (GRASSP). The GRASSP evaluation comprises four domains: muscle strength, sensory function, grasping ability, and prehension performance. In total, 129 sets of GRASSP evaluation data were used for the analysis. Measurements corresponding to areas of bodily function and structure were designated as ‘impairment’ measurements, while those relating to areas of activity were labeled as ‘task performance’ measurements. A random forest model was developed using ‘impairment’ measurements as input data and ‘task performance’ measurements as output data. The prediction model comprised 50 trees. Leave-one-out cross-validation was used to train the classifier and test its performance. The Spearman’s correlation coefficient between the predicted task performance scores and actual scores was 0.84. After removing the outliers, which accounted for 6.2% of the dataset, the coefficient increased to 0.92, indicating the high performance of the predictive model.

In 2019, McCoy et al. [33] recruited 47 patients with acute traumatic SCIs and developed a model utilizing a 2D CNN to segment the entire spinal cord and intramedullary spinal cord lesions using T2-weighted axial images of the cervical or thoracic spine. The model was based on the Brain and Spinal Cord Injury Center segmentation (BASICseg) network, which was further segmented into three variants: BASICseg-1 using dropout, BASICseg-2 using batch normalization, and BASICseg-3 using batch normalization and a noise adaptation layer. The segmentation outcomes of the BASICseg model were compared with those of the state-of-the-art methods PropSeg and DeepSeg. Performance assessments of the spinal cord and lesion segmentation were conducted using Dice coefficients. Additionally, segmented lesion volumes were employed in a linear regression analysis to determine their association with motor scores. Compared to manual labeling, the BASICseg model exhibited an average test set Dice coefficient of 0.93 for spinal cord segmentation, while PropSeg and DeepSeg achieved coefficients of 0.80 and 0.90, respectively. The BASICseg model demonstrated greater adaptability to lesion regions than PropSeg and DeepSeg. The volume of collision-related lesions based on automated segmentation were significantly associated with the motor scores upon admission (*p* = 0.002) and discharge (*p* = 0.009).

In 2022, Okimatsu et al. [34] developed a model to predict the American Spinal Cord Injury Association Impairment Scale (AIS) score using T2-weighted sagittal images of the cervical spinal cords of 215 individuals. They employed deep learning-based radiomics (DLR) to calculate the probabilities of AIS grades. In the MRI images, the region of interest was defined as the area encompassing the damaged segment of the spinal cord and the anterior and posterior boundaries at the injury level. The AIS grades were classified into five levels, ranging from the most severe grade, A (indicating the most serious injury), to the normal grade, E. Subsequently, an identification model was constructed using a random forest model based on three features: the probability of each AIS grade being obtained through DLR, age, and the initial AIS grade upon admission. The ensemble model based on DLR and random forest achieved an accuracy of 0.714, precision of 0.590, recall of 0.565, and an f1 score of 0.567. These results indicate the potential utility of combining DLR and random forest for predicting short-term neurological outcomes in acute cervical spinal cord injury. However, further refinement of the predictive model performance is necessary for practical clinical use. Efforts should be directed towards gathering data from a larger cohort of patients with cervical injuries to enhance accuracy. Additionally, potential confounding variables, such as blood pressure management or surgical intervention, could be considered. This study was conducted to investigate neurological prognosis one month post-injury; therefore, consideration should be given to developing algorithms for assessing long-term outcomes by collecting extended-term data.

The application of AI in predicting the prognosis of SCI is diverse, encompassing the development of models for identifying spinal cord lesions and predicting motor function prognosis. However, in the domain of predicting motor function, there are perceived limitations in practical applications in clinical settings.

## 4. Discussion

In this study, we investigated the integration of AI in predicting the outcome of CNS disorders. Active research has been aimed at utilizing AI to predict the prognosis of patients with stroke, TBI, and SCI. The application of AI algorithms is believed to assist in assessing the prognosis of patients with CNS disorders undergoing rehabilitation. However, the algorithms developed for specific medical conditions cannot be applied to other diseases. Therefore, it is necessary to develop AI algorithms for predicting the prognosis of various CNS disorders such as Parkinson’s disease, dementia, and multiple sclerosis, in addition to stroke, TBI, and SCI. Furthermore, to implement the AI algorithm effectively in real clinical scenarios, the accuracy of the predictions must be enhanced. To enhance the capabilities of AI algorithms, it is necessary to accumulate large volumes of data and integrate them from multiple medical institutions. Furthermore, previous studies have predominantly employed clinical data as the input. Given the proficiency of deep learning in analyzing image data, there is a need for research that employs imaging, such as MR and CT scans, to predict prognoses. However, there are several limitations to improving the prediction accuracy when applying AI algorithms in actual clinical settings. The availability of high-quality data is limited, and cooperation between institutions is essential for integrating data from multiple sources [3,44]. In addition, privacy concerns related to data usage can arise, making it crucial to securely anonymize and protect patient data [3]. The variability among patients can also pose challenges to the application of AI algorithms in clinical settings [44]. Given the wide range of patient responses to rehabilitation interventions, it is important to consider variability, because inaccurate predictions by AI algorithms can lead to serious problems. Considering these limitations, future research directions are suggested as follows: (1) Longitudinal tracking data can be highly useful in tracing recovery trajectories [45]; therefore, researchers should develop models capable of detecting changes over time. (2) Through international cooperation and data-sharing initiatives, researchers from different countries can access diverse datasets, towards facilitating the development of more robust and generalizable prognostic prediction models. (3) Multidisciplinary collaboration among ethics and policy experts, AI specialists, neuroscientists, clinicians, medical technicians, and healthcare professionals can promote a comprehensive understanding of CNS disorders. Moreover, sensitive privacy concerns related to patient data can be addressed. (4) Utilizing cutting-edge technologies, such as explainable AI, quantum computing, and advanced natural language processing, can facilitate the prognosis of CNS rehabilitation.

In the context of CNS rehabilitation, AI holds significant potential not only for prognosis prediction but also for pain management and complication prediction. Machine learning models can be used to identify pain-inducing factors and formulate personalized pain management strategies [46]. They can also be used to identify risk factors and predict and prevent complications associated with CNS disorders such as pressure sores and muscle contracture [47]. Furthermore, it is anticipated that AI-based prognostic prediction in CNS rehabilitation will significantly mitigate costs. Employing a targeted approach to identify patients who are most likely to benefit from specific rehabilitation interventions can lead to more effective treatment and faster recovery, which can reduce the overall treatment cost by minimizing the need for prolonged or unnecessary interventions. Another application of AI is resource allocation. Establishing examination and treatment plans based on a patient’s financial situation can ensure that patients receive the appropriate level of care without excessive resource consumption, thereby guaranteeing cost-effective care.

It is anticipated that as clinicians increasingly study and comprehend machine learning, AI will be further harnessed in the realm of CNS rehabilitation, paving the way for significant advancements in the future.

## Figures and Tables

**Table 1:** Characteristics of included studies.

Study	Target Data	Input Data	Output Data	Machine Learning Model	Outcome
Gupta et al. 2017 [23]	Demographic and clinical data of 575 patients with intracerebral hemorrhage	Demographic data, laboratory results, state at admission, treatment, neurological defects, hospital complications, medical history, and discharge data	Modified Rankin Scale for assessment of the degree of impairment or dependency in the daily activities of stroke patients	Random forest and linear regression	3 months exercise outcome prediction model: AUC of 0.89 12 months exercise outcome prediction model: AUC of 0.87
Heo et al. 2019 [24]	Demographic and clinical data of 2604 patients with ischemic stroke	Age, sex, smoking status, time from onset to admission, National Institutes of Health Stroke Scale scores, Trial of Org 10172 in Acute Stroke Treatment classification, Systolic and diastolic blood pressure, previous diseases, medication history, and laboratory values	Modified Rankin Scale score for assessment of the degree of impairment or dependency in the daily activities of stroke patients	Random forest, logistic regression, and deep neural network	Random forest: AUC of 0.857, sensitivity of 32.1%, and specificity of 98.4% Logistic regression: AUC of 0.849, sensitivity of 23.2%, and specificity of 98.9% Deep neural network: AUC of 0.888, sensitivity of 36.7%, and specificity of 98.4%
Lin et al. 2018 [25]	Demographic and clinical data of 313 patients with acute stroke	Age, modified Rankin Scale, Barthel index at admission, functional oral intake scale, mini nutrition assessment, European quality of life 5 dimensions questionnaire, instrumental activities of daily living scale, Berg balance test, gait speed, 6-min walk test, Fugl–Meyer upper extremity assessment, modified Fugl–Meyer sensory assessment, mini-mental state examination, motor activity log, and concise Chinese aphasia test	Barthel index for assessment of independence and mobility in daily life activities	Random forest, logistic regression, support vector machine	Random forest: AUC of 0.792, sensitivity of 65.0%, and specificity of 72.0% Logistic regression: AUC of 0.796, sensitivity of 72.7%, and specificity of 71.7% Support vector machine: AUC of 0.774, sensitivity of 67.7%, and specificity of 68.4%
Kim et al. 2022 [26]	Demographic and clinical data of 833 consecutive patients with stroke	Age, sex, type of stroke, and Medical Research Council score for muscle strength of the shoulder abductor, elbow flexor, finger flexor, finger extensor, hip flexor, knee extensor, and ankle dorsiflexor of the affected side	Modified Brunnstrom classification to evaluate upper extremity function and functional ambulation category to evaluate mobility function	Random forest, logistic regression, and deep neural network	Modified Brunnstrom classification prediction model - Random forest: AUC of 0.736 - Logistic regression: AUC of 0.790 - Deep neural network: AUC of 0.836 Functional ambulation category prediction model - Random forest: AUC of 0.741 - Logistic regression: AUC of 0.795 - Deep neural network: AUC of 0.0.836
Kim et al. 2021 [27]	Clinical data and MRI images of 221 patients with a corona radiata infarct	2-weighted axial images of corona radiata	Functional ambulation category to evaluate mobility function	Convolutional neural network	Prediction model included image data only: AUC of 0.751 Prediction model included clinical data and image data: AUC of 0.919
Shin et al. 2022 [28]	Clinical data and MRI images of 1233 patients with stroke	2-weighted axial images of brain	Modified Brunnstrom classification to evaluate upper extremity function and functional ambulation category to evaluate mobility function	Convolutional neural network	Modified Brunnstrom classification prediction model: AUC of 0.768, sensitivity of 71.36%, specificity of 71.14%, and precision of 78.5% Functional ambulation category prediction model: AUC of 0.828, sensitivity of 78.95%, specificity of 79.61%, and precision of 90.91%
Rizoli et al. 2016 [29]	Demographic and clinical data of 1089 patients with traumatic brain injury	Age, sex, systemic blood pressure, Head Abbreviated Injury Scale, Marshall score on the first head computed tomography, and pupil reactivity at emergency department admission	Glasgow Coma Scale to ascertain consciousness following brain injury	Decision tree	AUC of 0.67, sensitivity of 72.3%, and specificity of 62.5%
Gravesteijn et al. 2020 [30]	Demographic and clinical data of 11,022 patients with traumatic brain injury	Age, initial CT findings, presence of subarachnoid hemorrhage and hypoxia, and blood levels of glucose, sodium, and hemoglobin	Glasgow Coma Scale to ascertain consciousness following brain injury	Ridge regression, LASSO regression, random forest, logistic regression, gradient boosting machines, support vector machines, and neural network	Prediction model for mortality - Ridge regression: AUC of 0.82 - LASSO regression: AUC of 0.82 - Random forest: AUC of 0.81 - Logistic regression: AUC of 0.82 - Gradient boosting machines: AUC of 0.83 - Support vector machines: AUC of 0.81 - Neural network: AUC of 0.82 Prediction model for unfavorable outcome - Ridge regression: AUC of 0.77 - LASSO regression: AUC of 0.77 - Random forest: AUC of 0.76 - Logistic regression: AUC of 0.77 - Gradient boosting machines: AUC of 0.78 - Support vector machines: AUC of 0.78 - Neural network: AUC of 0.78
Matsuo et al. 2020 [31]	Demographic and clinical data of 232 patients with traumatic brain injury	Age, Glasgow Coma Scale score, abnormal pupillary response, systemic blood pressure, major extracranial injury, CT findings, and laboratory findings (glucose, C-reactive protein, and fibrin/fibrinogen degradation products)	Glasgow Outcome Scale to assess functional recovery after brain injury	Ridge regression, LASSO regression, random forest, gradient boosting, extra trees, decision trees, Gaussian naïve Bayes, multinomial naïve Bayes, and support vector machines (kernel consisted of linear, radial basis function, polynomial, and Sigmoid)	Morbidity prediction performance assessed using five-fold cross-validation - Ridge regression: AUC of 0.879, sensitivity of 88.2%, and specificity of 70.6% - LASSO regression: AUC of 0.863, sensitivity of 94.5%, and specificity of 48.3% - Random forest: AUC of 0.857, sensitivity of 97.2%, and specificity of 49.2% - Gradient boosting: AUC of 0.869, sensitivity of 93.7%, and specificity of 62.8% - Extra trees: AUC of 0.881, sensitivity of 95.8%, and specificity of 53.6% - Decision trees: AUC of 0.754, sensitivity of 87.5%, and specificity of 58.1% - Gaussian naïve Bayes: AUC of 0.842, sensitivity of 68.7%, and specificity of 82.8% - Multinomial naïve Bayes: AUC of 0.69, sensitivity of 83.2%, and specificity of 41.1% - Support vector machines(average): AUC of 0.882, sensitivity of 93.3%, and specificity of 57.2% Mortality prediction performance assessed using five-fold cross-validation - Ridge regression: AUC of 0.939, sensitivity of 85.1%, and specificity of 84.9% - LASSO regression: AUC of 0.776, sensitivity of 77.6%, and specificity of 91.7% - Random forest: AUC of 0.960, sensitivity of 64.4%, and specificity of 99.3% - Gradient boosting: AUC of 0.951, sensitivity of 73.8%, and specificity of 93.2% - Extra trees: AUC of 0.949, sensitivity of 68.0%, and specificity of 97.8% - Decision trees: AUC of 0.813, sensitivity of 68.5%, and specificity of 86.3% - Gaussian naïve Bayes: AUC of 0.890, sensitivity of 71.6%, and specificity of 89.4% - Multinomial naïve Bayes: AUC of 871, sensitivity of 66.4%, and specificity of 92.5% - Support vector machines(average): AUC of 0.917, sensitivity of 75.3%, and specificity of 95.2%
Zariffa et al. 2016 [32]	129 sets of GRASSP evaluation data	Data of impairment domain in GRASSP	Data of task performance domain in GRASSP	Random forest	The Spearman correlation coefficient between predicted task performance scores and actual scores was 0.92 after removing outlier data
Mccoy et al. 2019 [33]	MRI images of 47 patients with acute traumatic spinal cord injury	T2-weighted axial images of the cervical or thoracic spine	Spinal cord and lesion segmentation, and association with motor scores	Three models based on Brain and Spinal Cord Injury Center segmentation (basicseg) network	Dice coefficient of 0.93 for spinal cord segmentation There was a significant correlation between the size of collision-related lesions and motor scores at admission (*p* = 0.002) and discharge (*p* = 0.009) based on automatic segmentation
Okimatsu et al. 2022 [34]	MRI images of 215 patients with spinal cord injury	T2-weighted sagittal images of the cervical spinal cords	American Spinal Cord Injury Association Impairment Scale score to assess sensory and motor function	Ensemble model based on deep learning-based radiomics and random forest	0.714 of accuracy, 0.590 of precision, 0.565 of recall, and 0.567 of f1 score

Note: GRASSP, Graded Redefined Assessment of Strength, Sensibility, and Prehension; LASSO, least absolute shrinkage and selection operator.

## Data Availability

No new data were created or analyzed in this study. Data sharing is not applicable to this article.
